# Supplementary figures and images for: Pseudomonas intra-genus competition determines the protective function of synthetic bacterial communities in Arabidopsis thaliana
Source: PLoS Biol. 2025 Jul 15;23(7):e3002882. doi: 10.1371/journal.pbio.3002882 (PMC12262851; doi:10.1371/journal.pbio.3002882)

**A**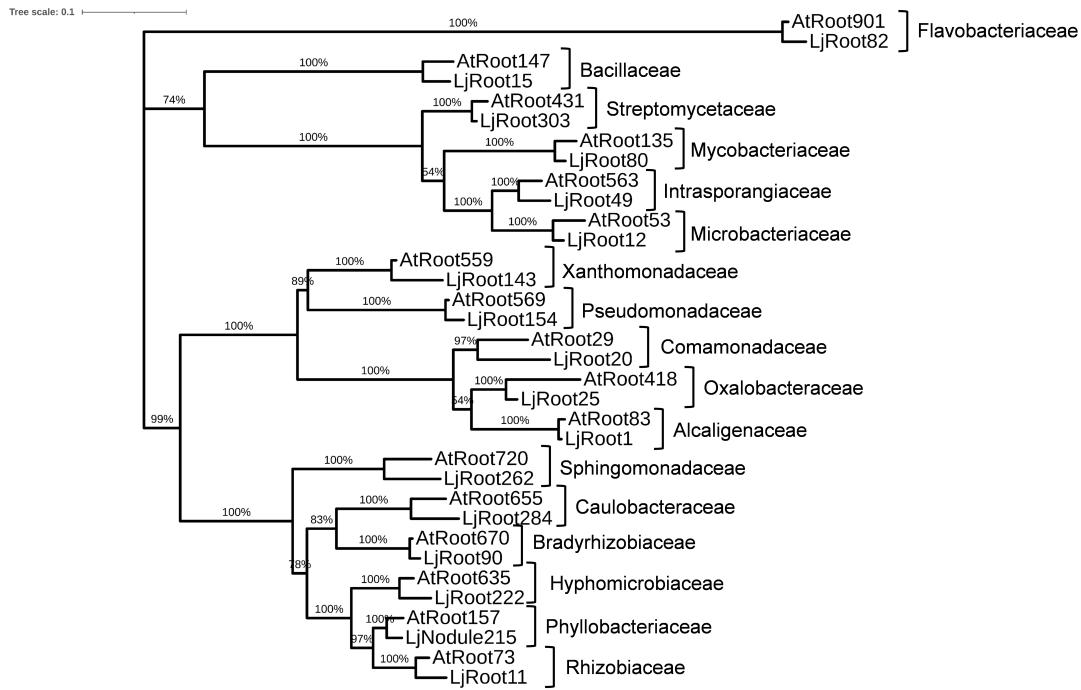**B**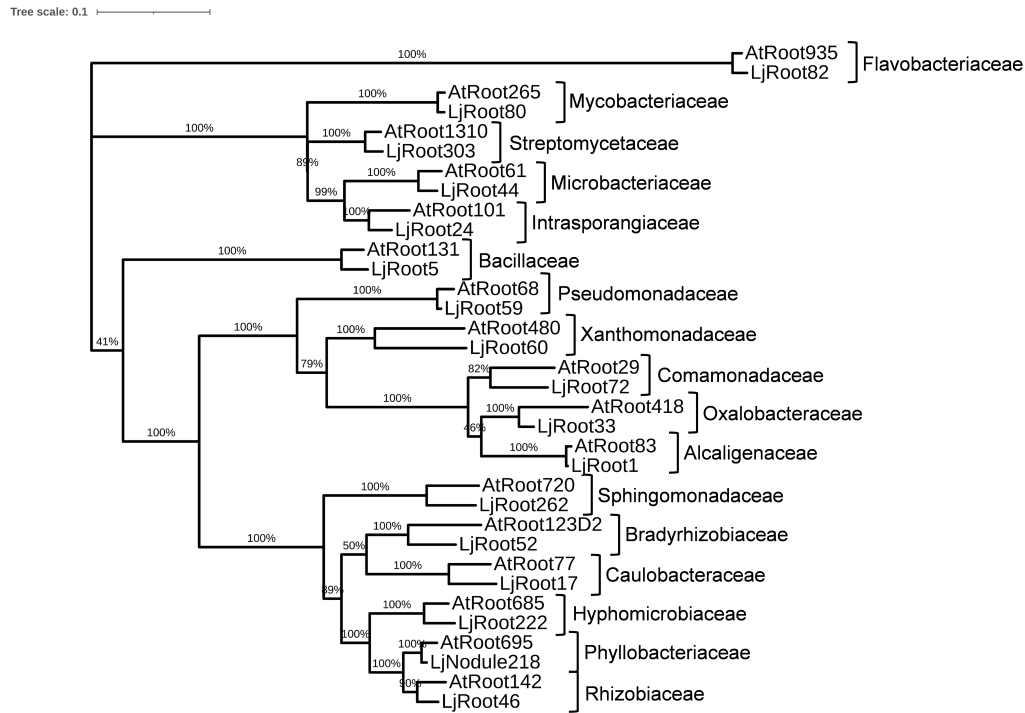

Supplement: S1 Fig — Tree is based on full-length 16S rRNA gene sequences of Lj and At SynCom strains (see Materials and Methods). Bootstrap number was 500. The SynComs were designed to exhibit high taxonomic diversity, and to represent the culturable root-associated community of A. thaliana and L. japonicus (i.e., one representative strain for each bacterial family shared between the At-RSPHERE and Lj-SPHERE collection; Wippel and colleagues 2021). The data underlying this figure can be found in S2 and S3 Files. (PDF) [file pbio.3002882.s001.pdf]

Dropped on the agar

AtRoot131

TY

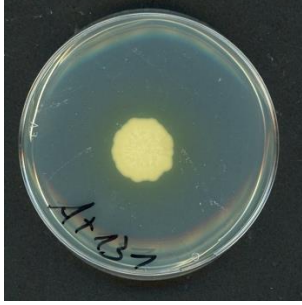

In the agar

TY+R401wt

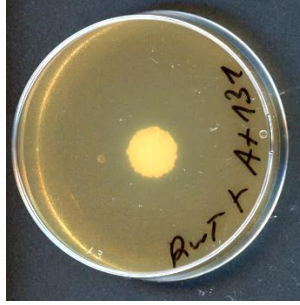

TY+R401mut

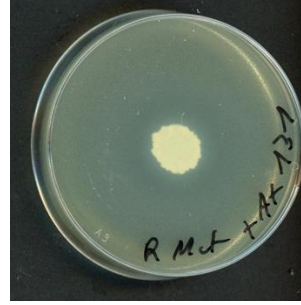

AtRoot68

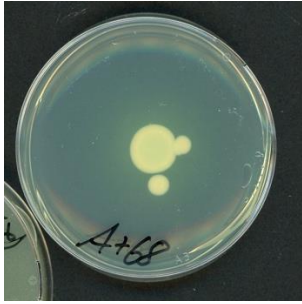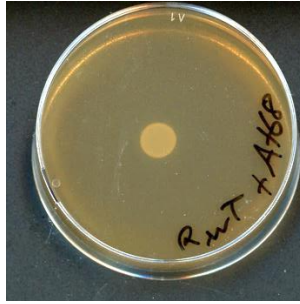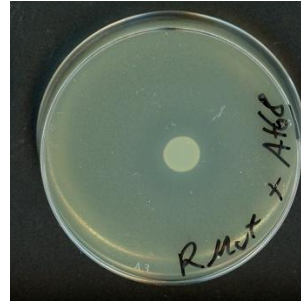

Supplement: S3 Fig — In vitro inhibition assay with AtRoot131 (upper row) and AtRoot68 (lower row) as producer. Left column, growth on TY medium only; middle column, growth on a bacterial lawn of R401wt, with slightly visible inhibition halo formed around the AtRoot131 colony; right column, growth on a bacterial lawn of R401mut, with well visible inhibition halo. (PDF) [file pbio.3002882.s003.pdf]

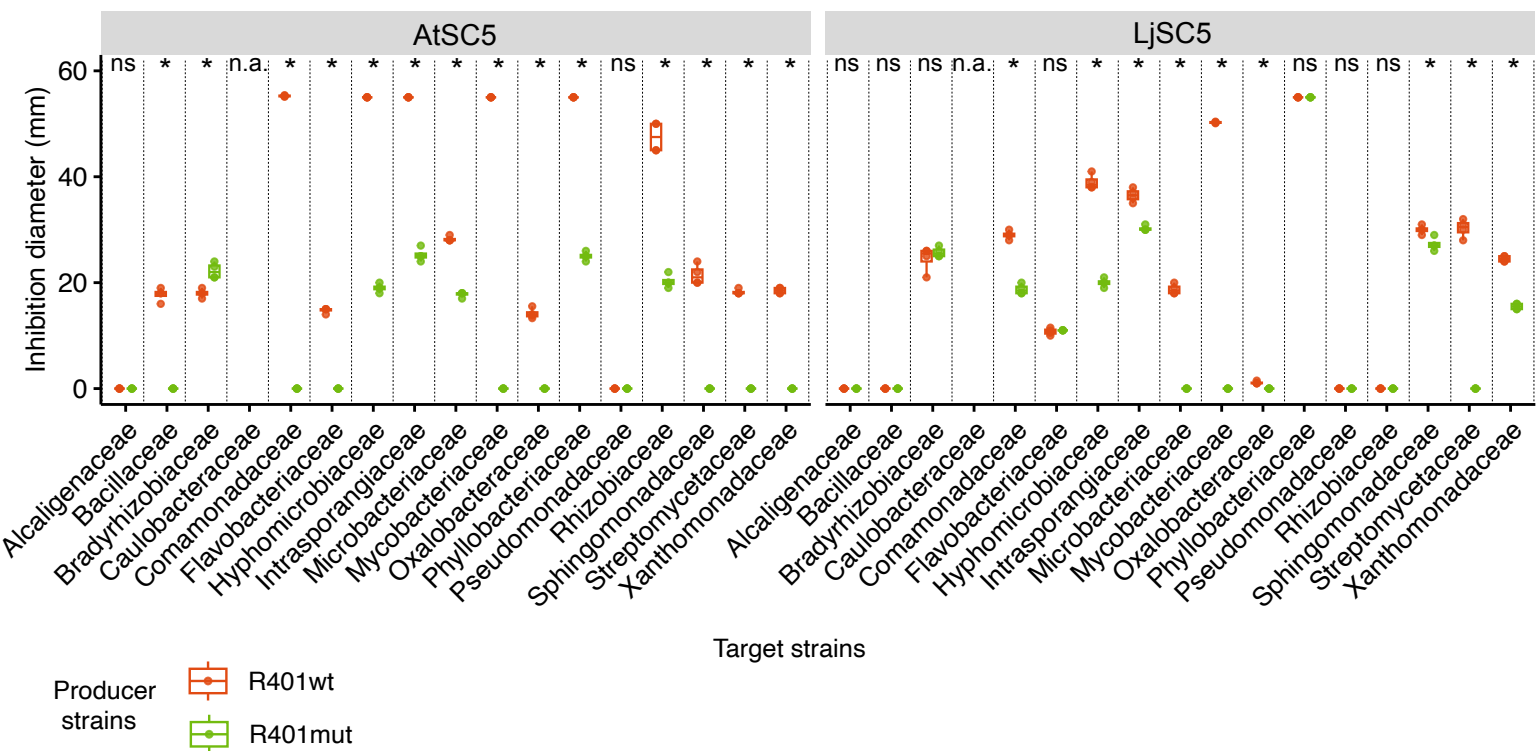

Supplement: S4 Fig — Inhibition diameter formed around either a R401wt or a R401mut colony (producers) on a lawn of the indicated individual commensal strains (targets) on an agar plate. Asterisks indicate significant (p < 0.05) difference between diameters caused by R401wt and R401mut according to Wilcoxon rank sum test. The data underlying this figure can be found in S1 Data tab S4. (PDF) [file pbio.3002882.s004.pdf]

**A**32.8 % of variance;  $p=0.001$ 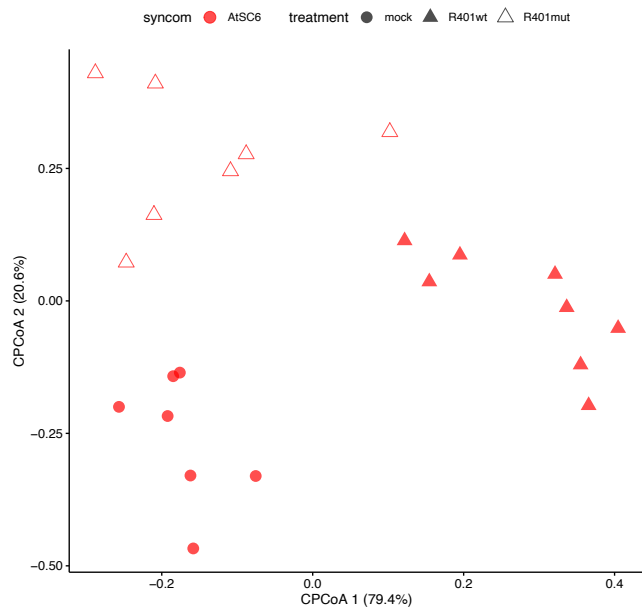**B**12.4 % of variance;  $p=0.12$ 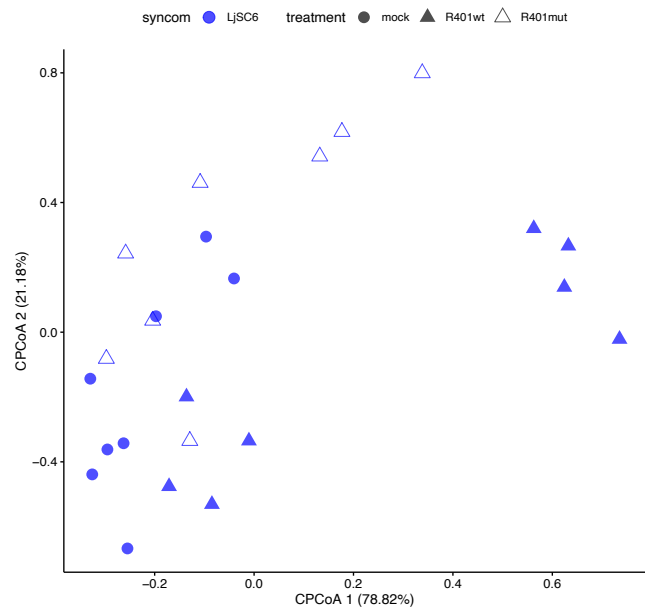

Supplement: S5 Fig — Beta-diversity of (A) At and (B) Lj SynComs after community establishment on Col-0 roots in the agar-based growth setup. Constrained Principal Coordinate Analyses (CPCoA; constrained by treatment) are shown for Bray-Curtis dissimilarities between 16S rRNA gene amplicons amplified from root samples of three-week old Col-0 plants that had been co-cultivated with the SynComs for two weeks, then infected with mock, R401wt, or R401mut, and grown for another week. Variance explained by infection is 32.8% (PERMANOVA, p = 0.001) and 12.4% (p = 0.12) for AtSC6- and LjSC6-co-cultivated plants, respectively. The data underlying this figure can be found in S1 Data tabs S5A and S5B. (PDF) [file pbio.3002882.s005.pdf]

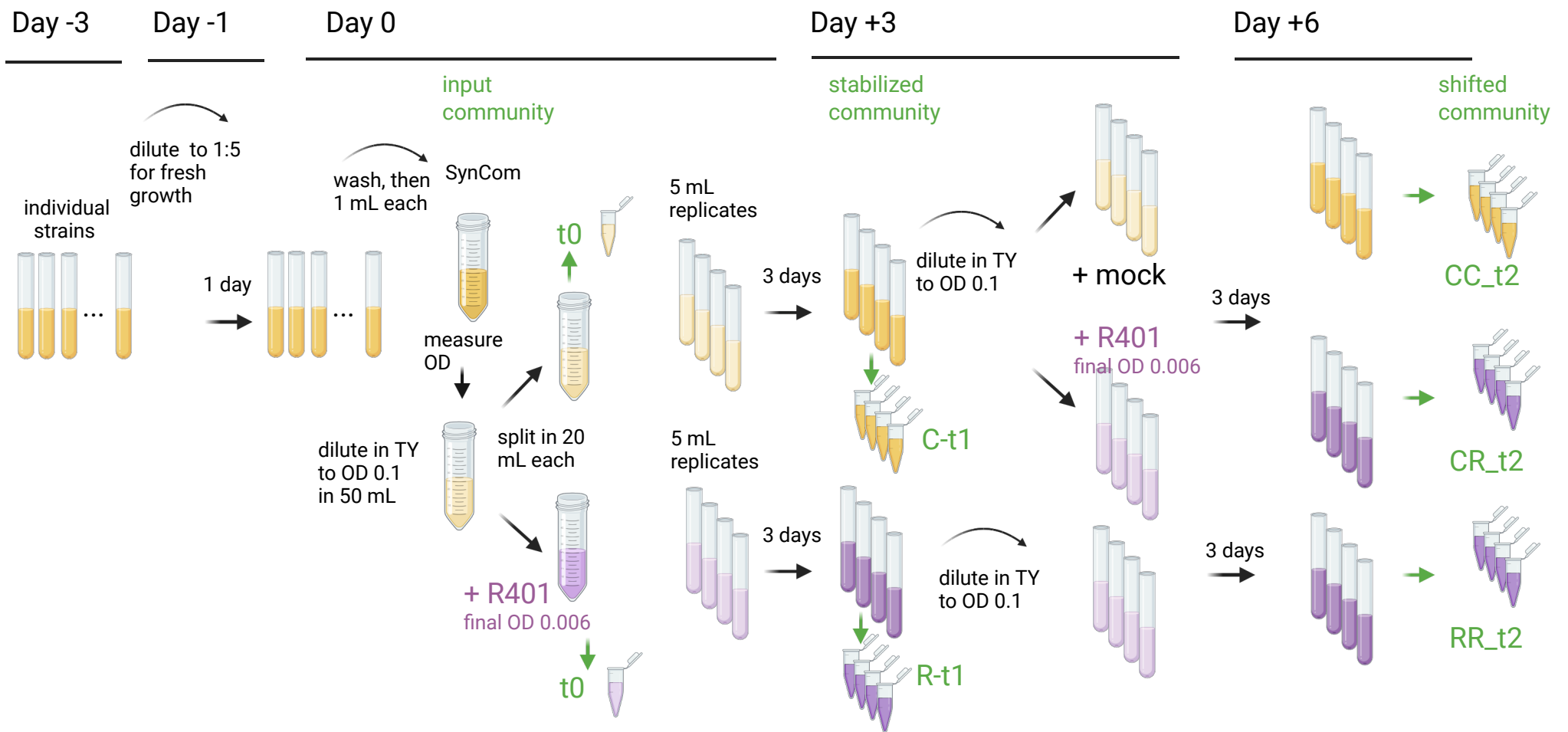

Supplement: S6 Fig — Cultivation of commensal strains with or without R401wt, and time points of sampling. See Methods sections for details. Created in BioRender. Wippel, K. (2025) https://BioRender.com/i0y55bb. (PDF) [file pbio.3002882.s006.pdf]

**A**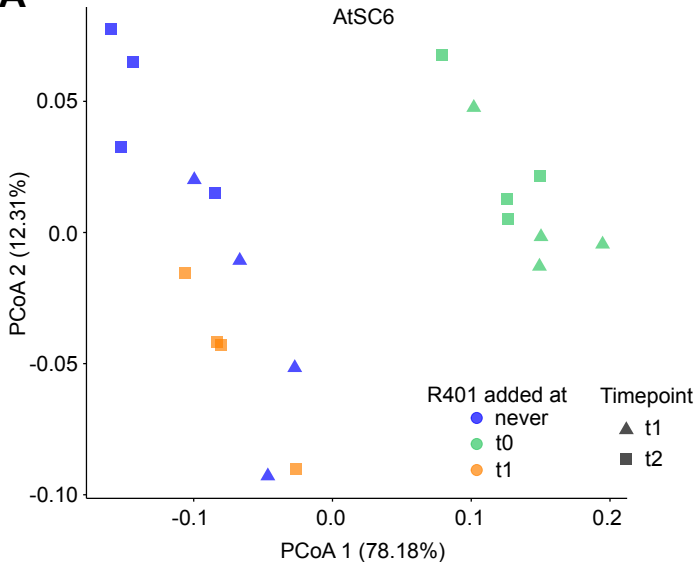**B**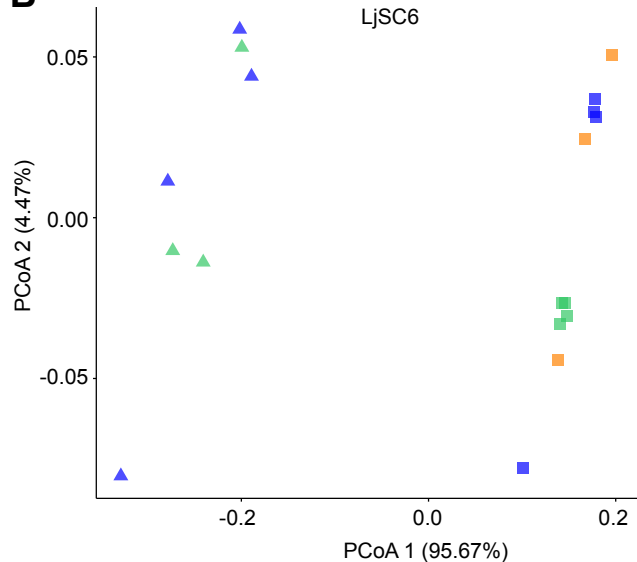

Supplement: S7 Fig — (A and B), Principal Coordinate Analysis (PCoA) of Bray-Curtis dissimilarities of 16S rRNA gene amplicons of liquid culture samples from SynComs (A) AtSC6 and (B) LjSC6. SynComs were grown in liquid medium for three days with or without R401wt (green and blue symbols, respectively). Then R401wt was added or not to the initially non-treated samples (orange and blue symbols, respectively). Samples were taken and profiled after three (t1) and after six days (t2). See S6 Fig for detailed experimental setup. The data underlying this figure can be found in S1 Data tabs S7A and S7B. (PDF) [file pbio.3002882.s007.pdf]

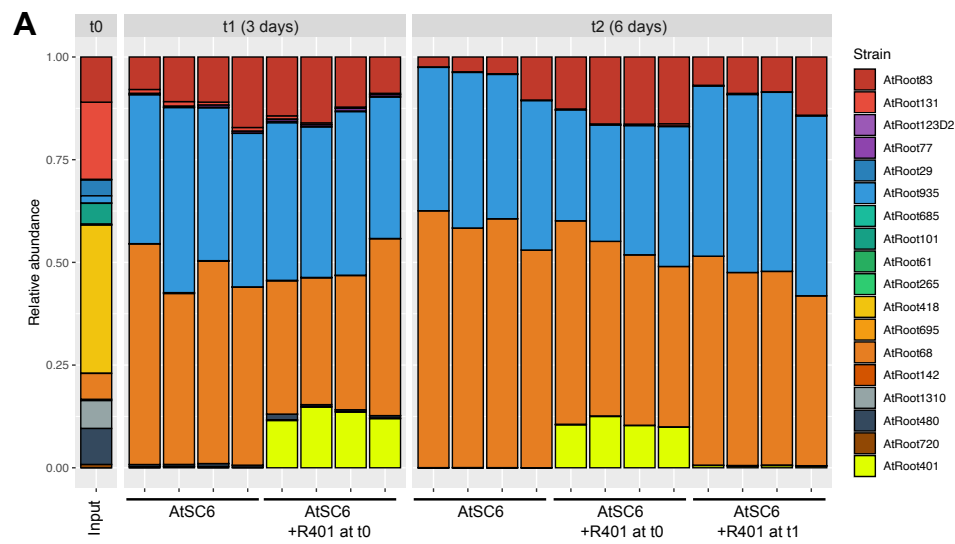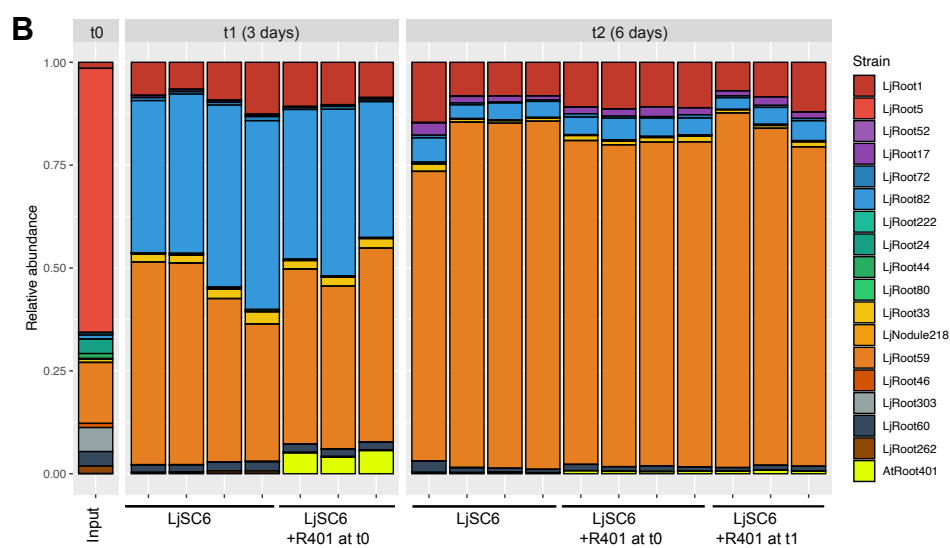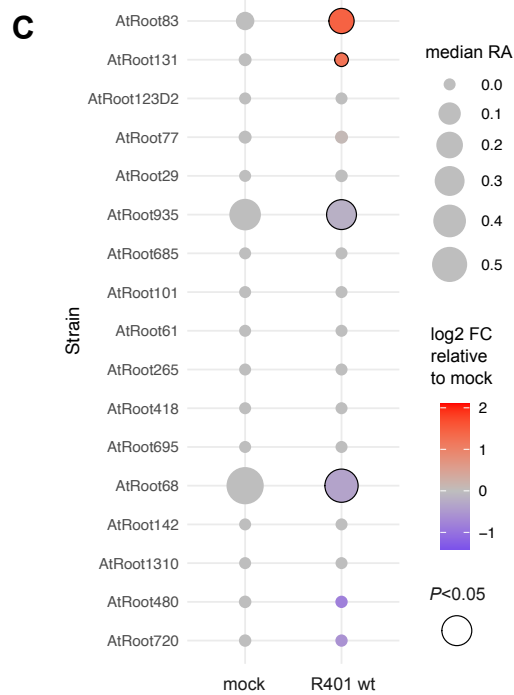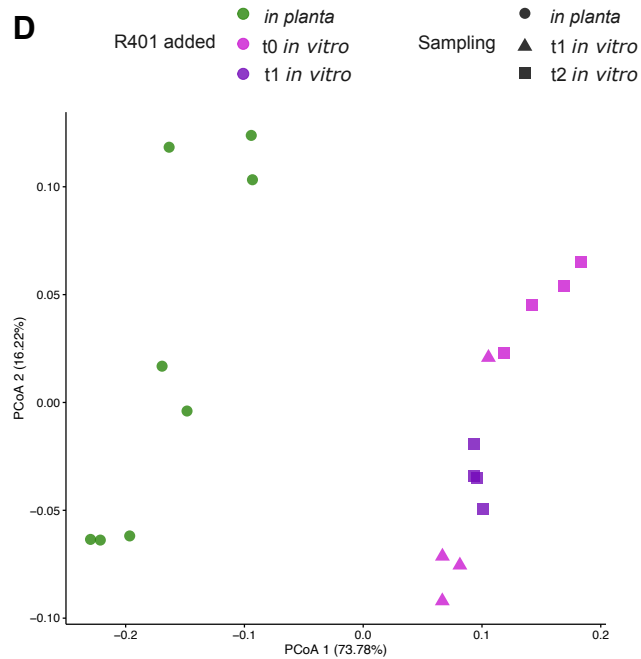

Supplement: S8 Fig — Relative abundance (RA) of individual commensal bacterial strains within (A) AtSC6 and (B) LjSC6 after growth for three (t1) and six (t2) days in liquid culture with or without R401wt added from the start or after three days, based on 16S rRNA amplicon reads. The composition of the communities at the start of the experiment is shown as t0. C, Quantification of individual bacterial strains within SynCom AtSC6 after six days (t2). Circle size corresponds to median relative abundance (RA). Color gradient indicates log2-transformed fold change of abundance relative to mock condition, where R401 was not added at t0 (gray indicates no change). Black circle outline indicates significant fold change (Wilcoxon rank sum test, p < 0.05). Strain IDs are indicated. D, Principal Coordinate Analysis (PCoA) of Bray-Curtis dissimilarities of 16S rRNA gene amplicons from AtSC6 of liquid culture samples (magenta symbols) or root-associated samples (green symbols) from the in planta experiment (Fig 2B), in presence of R401. Shapes depict samples from liquid cultures after three days (t1, triangles) and after six days (t2, squares). The data underlying this figure can be found in S1 Data tabs S8A, S8B, S8C, and S8D. (PDF) [file pbio.3002882.s008.pdf]

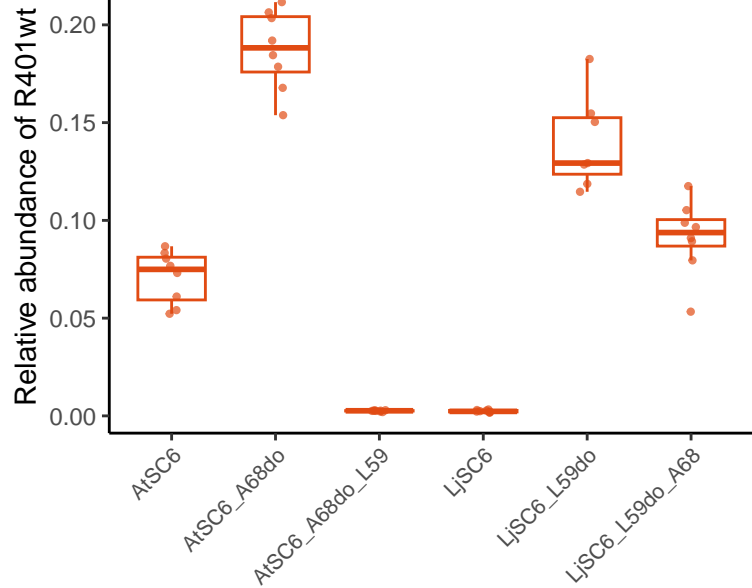

Supplement: S9 Fig — Relative abundance based on 16S rRNA gene amplicon reads of R401wt within the indicated SynComs established on roots of three-week old Col-0 plants that had been co-cultivated with the SynComs for two weeks, then infected with R401wt, and grown for another week. AtSC6_A68do (do, “dropout”), AtSC6 without AtRoot68; AtSC6_A68do_L59, AtSC6 without AtRoot68 but with LjRoot59; LjSC6_L59do, LjSC6 without LjRoot59; LjSC6_L59do_A68, LjSC6 without LjRoot59 but with AtRoot68. The data underlying this figure can be found in S1 Data tab S9. (PDF) [file pbio.3002882.s009.pdf]

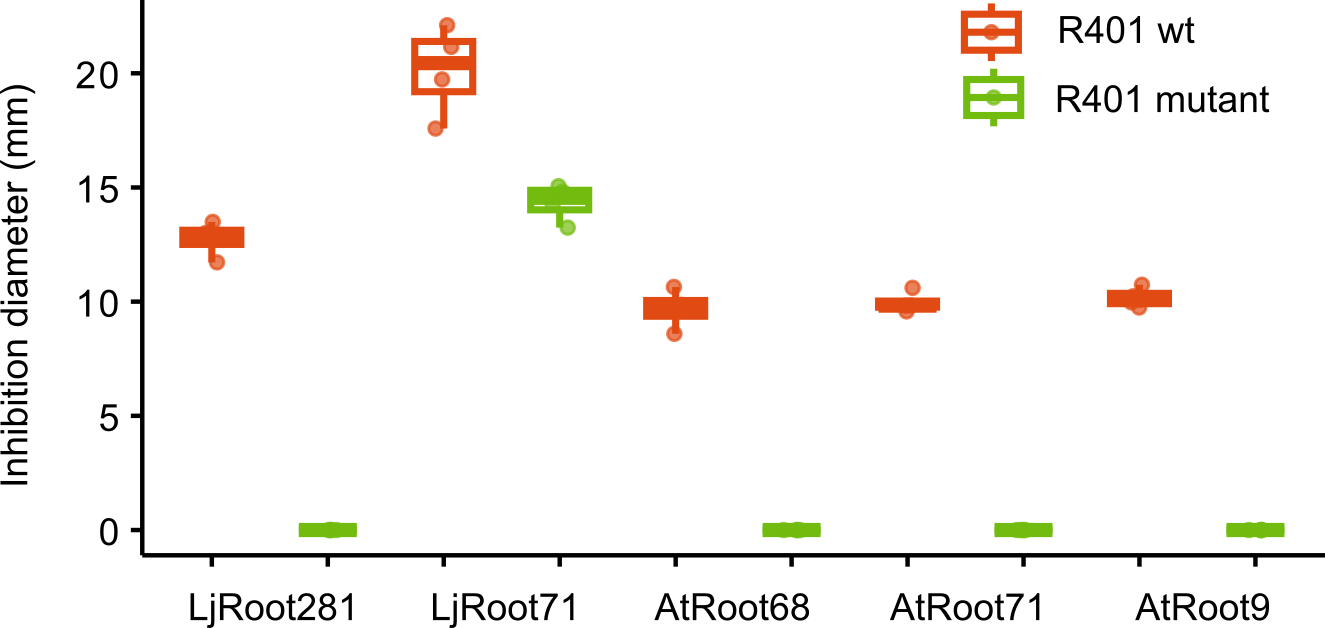

Supplement: S10 Fig — The inhibition diameter of the halo inhibition assay with either R401wt or R401mut as producers and the Pseudomonas commensals as targets is shown. The other 10 isolates were not inhibited. The data underlying this figure can be found in S1 Data tab S10. (TIF) [file pbio.3002882.s010.tif]

**A**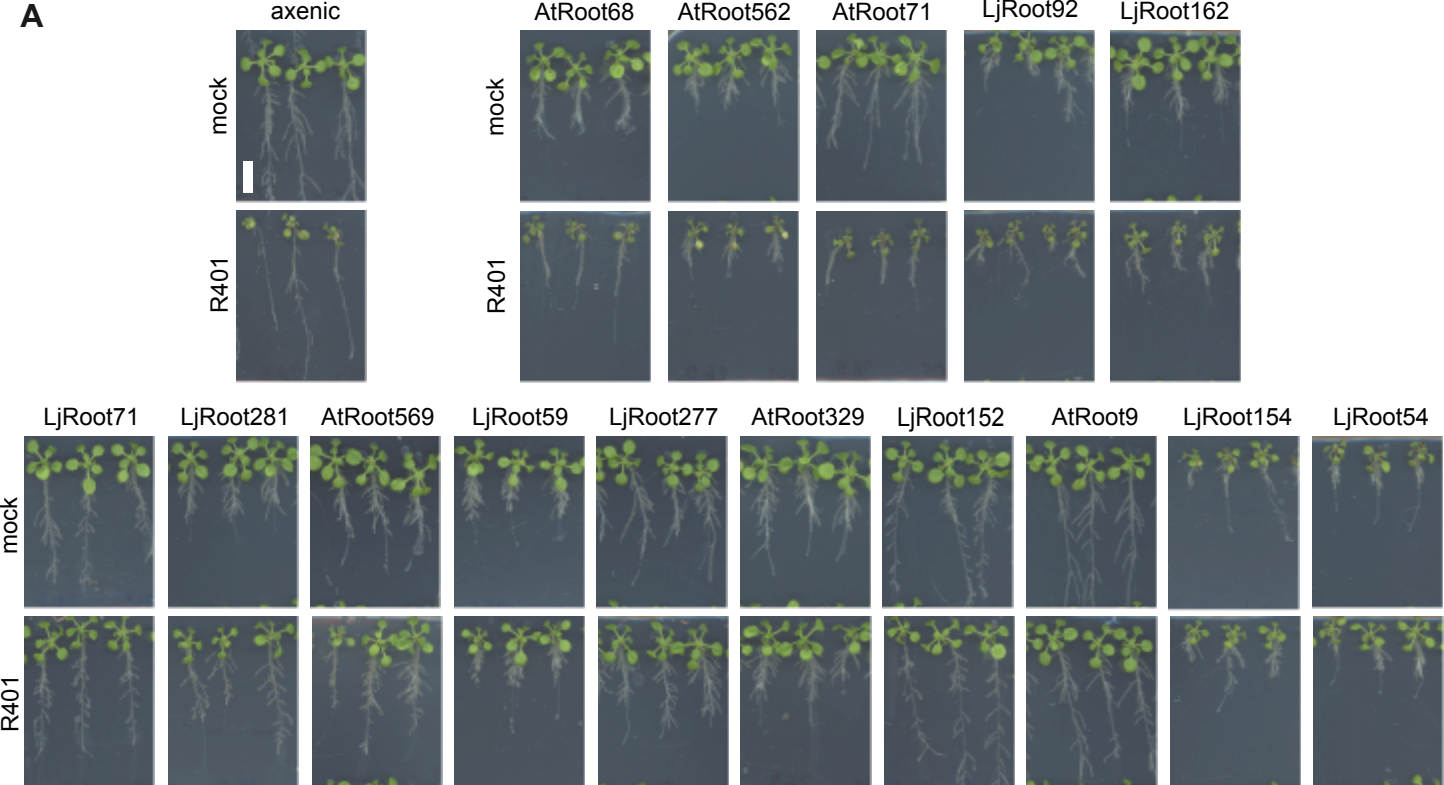**B**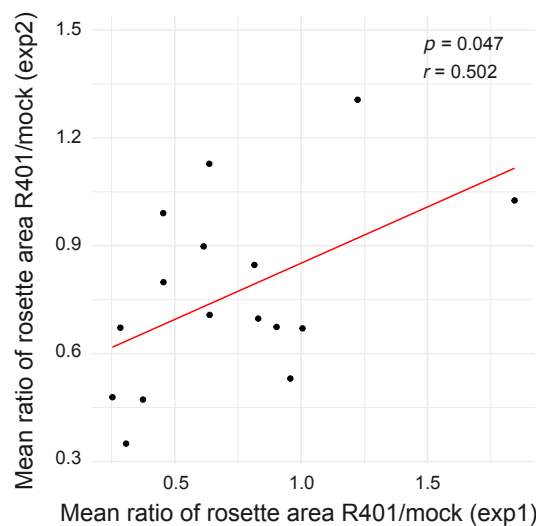

Supplement: S11 Fig — A, Col-0 phenotypes in co-cultivation with the indicated individual Pseudomonas strains after treatment with mock or R401wt. Scale bar corresponds to 1 cm. Images follow the order of the strain IDs in Fig 4B. B, Correlation between mean ratio values of rosette area of R401-treated relative to mock-treated plants from experiments performed in Cologne (exp1) and in Amsterdam (exp2); Pearson’s correlation coefficient r and p-value are shown. The data underlying this figure can be found in S1 Data tab S11B. (PDF) [file pbio.3002882.s011.pdf]

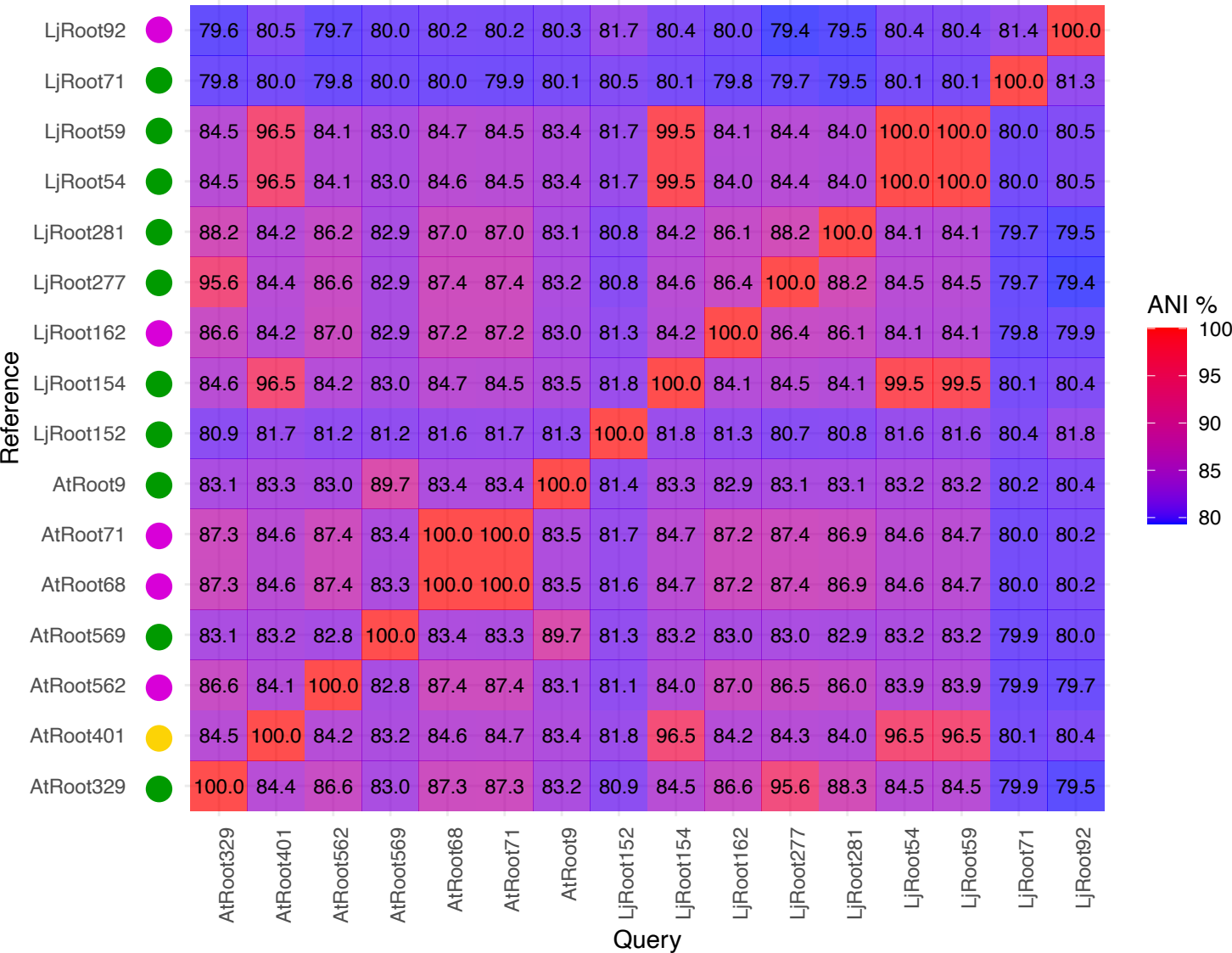

Supplement: S12 Fig — ANI is shown as percentage similarity between each pair of strains. Filled circles indicate protection (green) and no protection (magenta) of Col-0 by these strains against R401wt when in binary in planta assays (Fig 4). (PDF) [file pbio.3002882.s012.pdf]

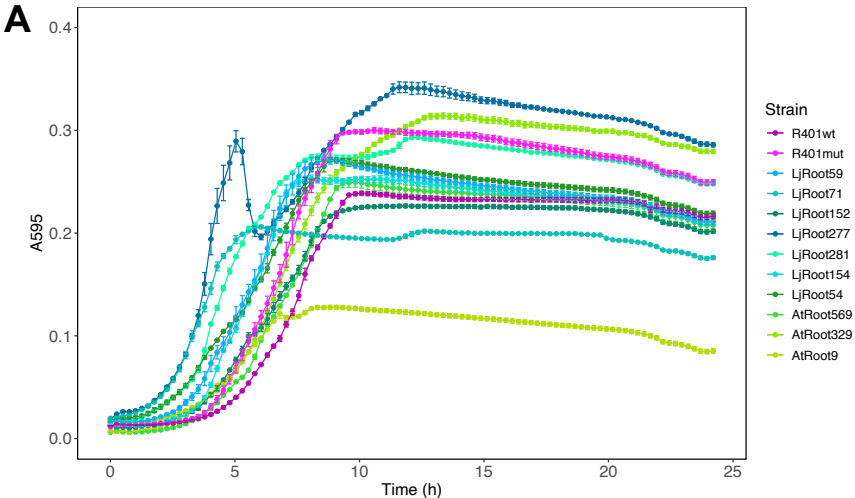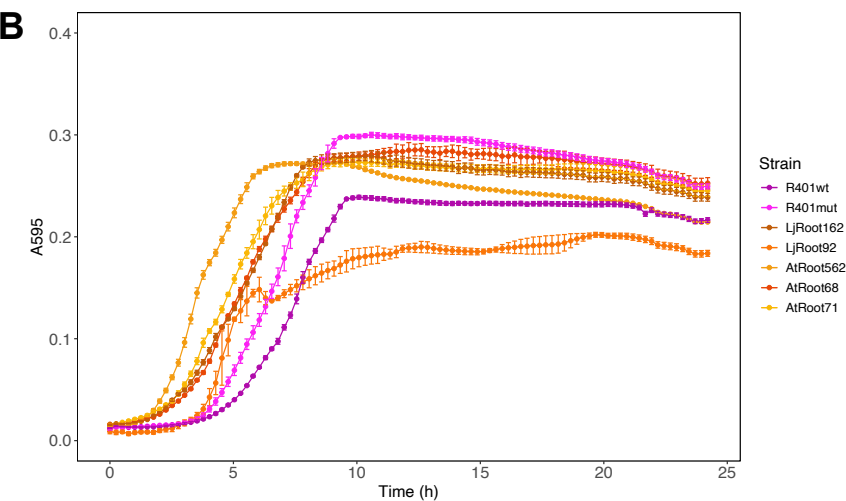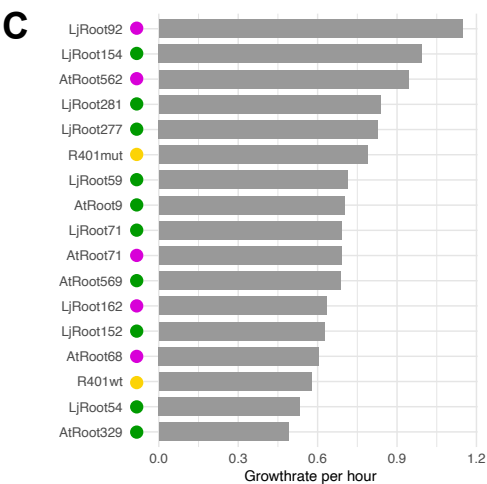

Supplement: S13 Fig — (A and B) Growth curves of indicated Pseudomonas isolates in minimal medium supplemented with ARE (artificial root exudates: glucose, fructose, sucrose, citric acid, succinate, lactate, glutamate, alanine, and serine). (A) Strains that protect plants against R401wt. (B) Strains that fail to protect plants against R401wt. (C) Corresponding growth rates. Color code next to the strain ID indicates protective activity against R401wt in planta. Filled circles indicate protection (green) and no protection (magenta) of Col-0 by these strains against R401wt when in binary in planta assays. The data underlying this figure can be found in S1 Data tabs S13AB and S13C. (PDF) [file pbio.3002882.s013.pdf]

Number  
of CGCs

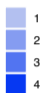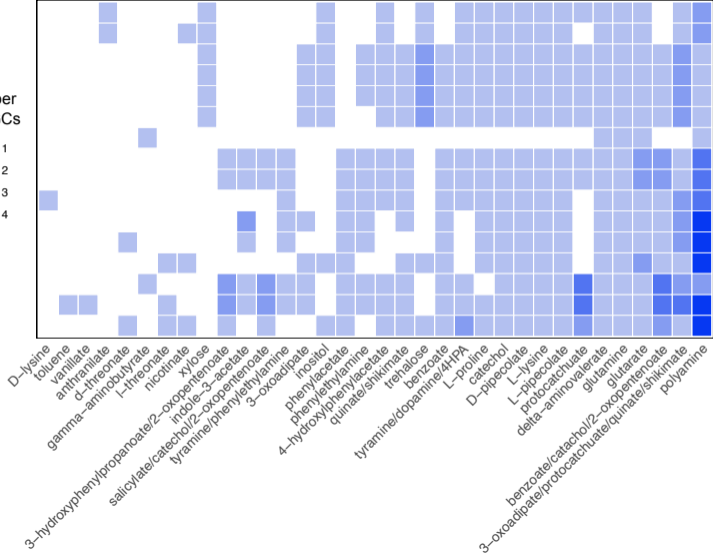

Supplement: S14 Fig — Number of catabolic gene clusters (CGCs) related to the catabolism of the indicated compounds in individual Pseudomonas strains, based on a survey of their whole-genome sequences using rhizoSMASH (see Materials and methods). The dendrogram shows the hierarchical clustering of the strains based on a distance matrix of the number of CGCs within each strain. Filled circles indicate protection (green) and no protection (magenta) of Col-0 by these strains against R401wt when in binary in planta assays (see Fig 4). The data underlying this figure can be found in S6 Table. (PDF) [file pbio.3002882.s014.pdf]

Heatmap of Euclidean Distances Between Strains

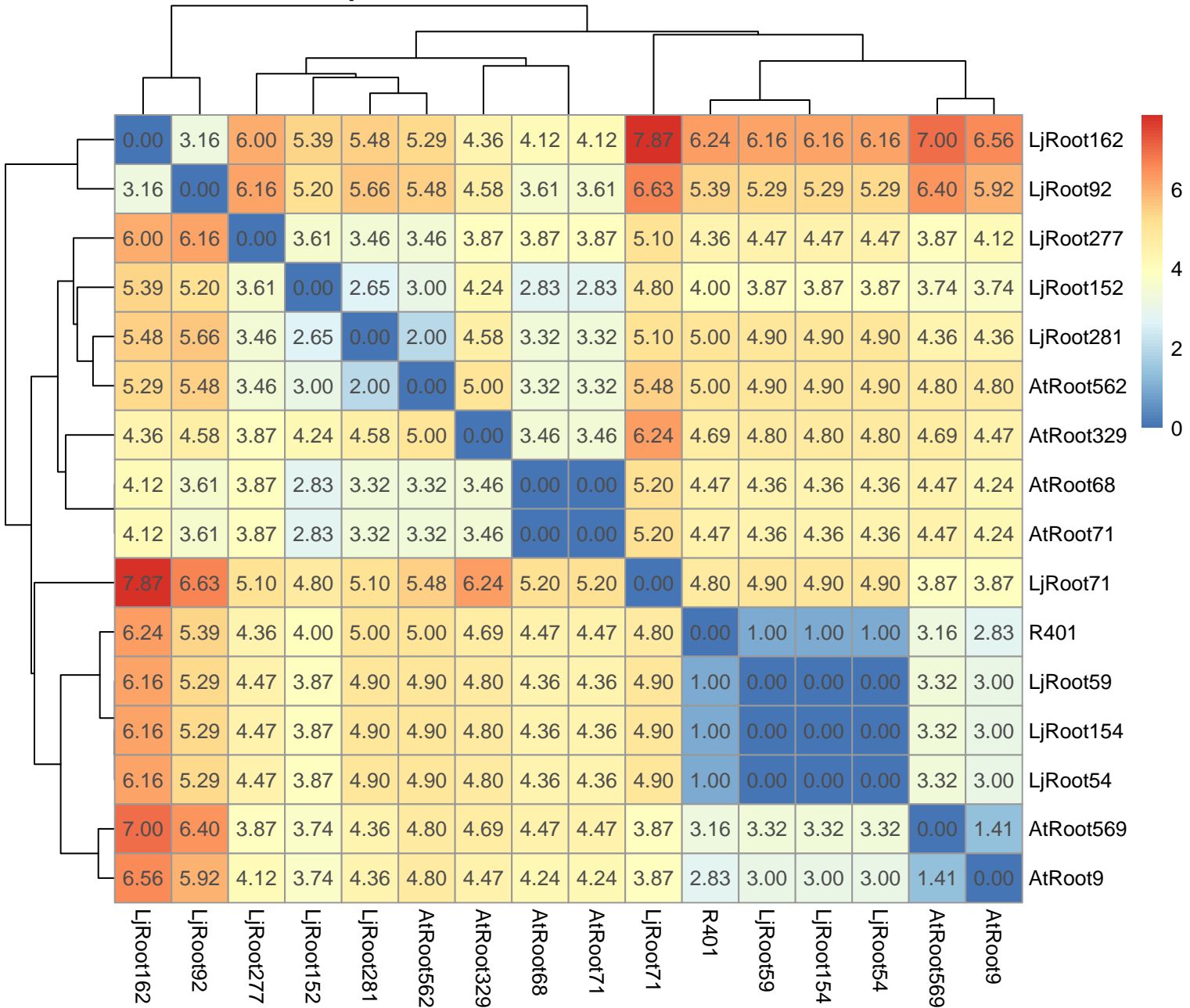

Supplement: S15 Fig — Based on the type and number of catabolic gene clusters (CGCs) detected in the bacterial whole-genome sequences. Distances are shown as numbers within the plot. The data underlying this figure can be found in S7 Tables. (PDF) [file pbio.3002882.s015.pdf]
