# Supplementary material for: Pseudomonas intra-genus competition determines the protective function of synthetic bacterial communities in Arabidopsis thaliana
Source: PLoS Biol. 2025 Jul 15;23(7):e3002882. doi: 10.1371/journal.pbio.3002882 (PMC12262851; doi:10.1371/journal.pbio.3002882)
Supplement: S2 Fig — See Methods section for details. Created in BioRender. Wippel, K. (2025) https://BioRender.com/i0y55bb. (PDF) [file pbio.3002882.s002.pdf]

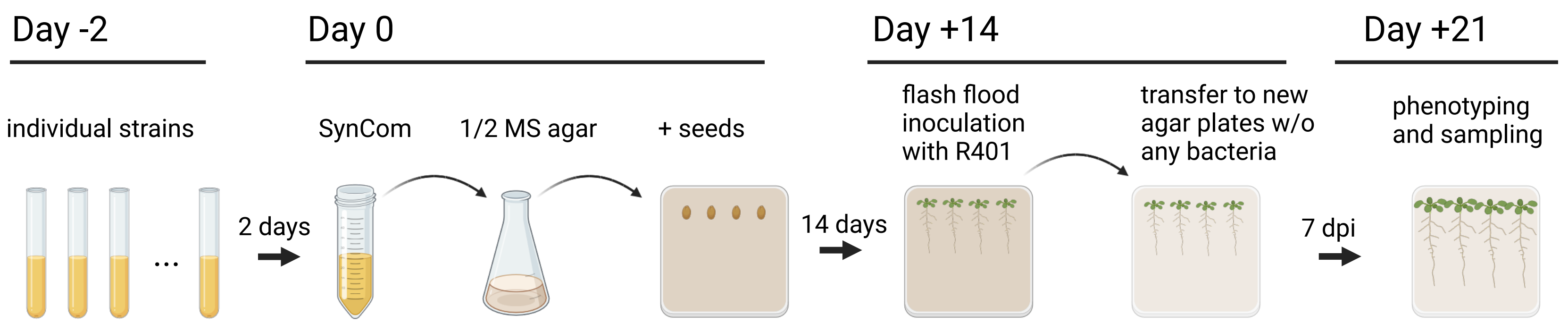
Day -2
Day 0
Day +14
Day +21
individual strains
...
2 days
SynCom
1/2 MS agar
+ seeds
14 days
flash flood
inoculation
with R401
transfer to new
agar plates w/o
any bacteria
7 dpi
phenotyping
and sampling
