# Supplementary material for: Pseudomonas intra-genus competition determines the protective function of synthetic bacterial communities in Arabidopsis thaliana
Source: PLoS Biol. 2025 Jul 15;23(7):e3002882. doi: 10.1371/journal.pbio.3002882 (PMC12262851; doi:10.1371/journal.pbio.3002882)
Supplement: S1 Table — (PDF) [file pbio.3002882.s016.pdf]

S1 Table: SynCom strains.

Strain IDs are according to Bai et al. 2015 [1] and Wippel et al. 2021 [2]. All strains are usually grown in 50% TSB medium, the ones highlighted grow better in TY+CaCl2.

Class	Order	Family	AtSC5	LjSC5	AtSC6	LjSC6
Betaproteobacteria	Burkholderiales	Alcaligenaceae	AtRoot83	LjRoot1	AtRoot83	LjRoot1
Bacillota (Firmicutes)	Bacillales	Bacillaceae	AtRoot147	LjRoot15	AtRoot131	LjRoot5
Alphaproteobacteria	Rhizobiales	Bradyrhizobiaceae	AtRoot670	LjRoot90	AtRoot123D2	LjRoot52
Alphaproteobacteria	Caulobacterales	Caulobacteraceae	AtRoot655	LjRoot284	AtRoot77	LjRoot17
Betaproteobacteria	Burkholderiales	Comamonadaceae	AtRoot29	LjRoot20	AtRoot29	LjRoot72
Bacteroidota (Bacteroidetes)	Flavobacteriales	Flavobacteriaceae	AtRoot901	LjRoot82	AtRoot935	LjRoot82
Alphaproteobacteria	Rhizobiales	Hyphomicrobiaceae	AtRoot635	LjRoot222	AtRoot685	LjRoot222
Actinomycetota (Actinobacteria)	Actinomycetales	Intrasporangiaceae	AtRoot563	LjRoot49	AtRoot101	LjRoot24
Actinomycetota (Actinobacteria)	Actinomycetales	Microbacteriaceae	AtRoot53	LjRoot12	AtRoot61	LjRoot44
Actinomycetota (Actinobacteria)	Actinomycetales	Mycobacteriaceae	AtRoot135	LjRoot80	AtRoot265	LjRoot80
Betaproteobacteria	Burkholderiales	Oxalobacteraceae	AtRoot418	LjRoot25	AtRoot418	LjRoot33
Alphaproteobacteria	Rhizobiales	Phyllobacteriaceae	AtRoot157	LjNodule215	AtRoot695	LjNodule218
Gammaproteobacteria	Pseudomonadales	Pseudomonadaceae	AtRoot569	LjRoot154	AtRoot68	LjRoot59
Alphaproteobacteria	Rhizobiales	Rhizobiaceae	AtRoot73	LjRoot11	AtRoot142	LjRoot46
Alphaproteobacteria	Sphingomonadales	Sphingomonadaceae	AtRoot720	LjRoot262	AtRoot720	LjRoot262
Actinomycetota (Actinobacteria)	Actinomycetales	Streptomycetaceae	AtRoot431	LjRoot303	AtRoot1310	LjRoot303
Gammaproteobacteria	Xanthomonadales	Xanthomonadaceae	AtRoot559	LjRoot143	AtRoot480	LjRoot60

References:

1. Bai Y et al. Functional overlap of the Arabidopsis leaf and root microbiota. *Nature* 2015;**528**:364–369. https://doi.org/10.1038/nature16192
2. Wippel K et al. Host preference and invasiveness of commensal bacteria in the Lotus and Arabidopsis root microbiota. *Nat Microbiol* 2021;**6**:1150–1162. https://doi.org/10.1038/s41564-021-00941-9
