# Supplementary material for: Pseudomonas intra-genus competition determines the protective function of synthetic bacterial communities in Arabidopsis thaliana
Source: PLoS Biol. 2025 Jul 15;23(7):e3002882. doi: 10.1371/journal.pbio.3002882 (PMC12262851; doi:10.1371/journal.pbio.3002882)
Supplement: S2 Table — (PDF) [file pbio.3002882.s017.pdf]

S2 Table: ***Pseudomonas*** **strains used in this study.**
Strain IDs are according to Bai et al. 2015 [1] and Wippel et al. 2021 [2].

AtRoot9
AtRoot68
AtRoot71
AtRoot329
AtRoot562
AtRoot569
LjRoot54
LjRoot59
LjRoot71
LjRoot92
LjRoot152
LjRoot154
LjRoot162
LjRoot277
LjRoot281

References:
1. Bai Y et al. Functional overlap of the Arabidopsis leaf and root microbiota. *Nature* 2015;**528**:364–369. https://doi.org/10.1038/nature16192
2. Wippel K et al. Host preference and invasiveness of commensal bacteria in the Lotus and Arabidopsis root microbiota. *Nat Microbiol* 2021;**6**:1150–1162. https://doi.org/10.1038/s41564-021-00941-
