# Supplementary material for: Pseudomonas intra-genus competition determines the protective function of synthetic bacterial communities in Arabidopsis thaliana
Source: PLoS Biol. 2025 Jul 15;23(7):e3002882. doi: 10.1371/journal.pbio.3002882 (PMC12262851; doi:10.1371/journal.pbio.3002882)
Supplement: S3 Table — (PDF) [file pbio.3002882.s018.pdf]

S3 Table: Distance indices of commensal *Pseudomonas* strains to Root401, based on distance matrix of full-length 16S rRNA gene sequence.
Protection is based on the plant phenotype after co-cultivation with individual strains and infection with R401wt.

strain	distance	protection
LjRoot92	0.20974299	FALSE
LjRoot71	0.18119034	TRUE
AtRoot329	0.1025641	TRUE
LjRoot277	0.12280866	TRUE
AtRoot569	0.13789994	TRUE
AtRoot9	0.14025737	TRUE
LjRoot54	0.0362143	TRUE
LjRoot59	0.0362143	TRUE
LjRoot154	0.0362143	TRUE
LjRoot281	0.09917694	TRUE
LjRoot152	0.16484512	TRUE
AtRoot68	0.12881971	FALSE
AtRoot71	0.12280866	FALSE
LjRoot162	0.15795808	FALSE
AtRoot562	0.15223061	FALSE

Pearson's product-moment correlation

data: distances and protection
t = -1.8163, df = 13, p-value = 0.09244
alternative hypothesis: true correlation is not equal to 0
95 percent confidence interval:
 -0.78194883 0.08104104
sample estimates:
       cor
 -0.4498991
