# Supplementary material for: Pseudomonas intra-genus competition determines the protective function of synthetic bacterial communities in Arabidopsis thaliana
Source: PLoS Biol. 2025 Jul 15;23(7):e3002882. doi: 10.1371/journal.pbio.3002882 (PMC12262851; doi:10.1371/journal.pbio.3002882)
Supplement: S4 Table — (PDF) [file pbio.3002882.s019.pdf]

S4 Table: Results of FastANI analysis of *Pseudomonas* genomes.

query	reference	ANI	fragments_aligned	total_fragments
AtRoot569	AtRoot569	100	2060	2060
AtRoot569	AtRoot9	89.7268	1673	2060
AtRoot569	AtRoot71	83.393	1133	2060
AtRoot569	AtRoot68	83.3285	1136	2060
AtRoot569	AtRoot401	83.1703	1178	2060
AtRoot569	LjRoot54	83.0481	1196	2060
AtRoot569	LjRoot59	83.0352	1199	2060
AtRoot569	AtRoot329	82.9984	1200	2060
AtRoot569	LjRoot154	82.9861	1205	2060
AtRoot569	LjRoot277	82.9428	1209	2060
AtRoot569	LjRoot281	82.9338	1126	2060
AtRoot569	LjRoot162	82.9074	1124	2060
AtRoot569	AtRoot562	82.8366	1111	2060
AtRoot569	LjRoot152	81.1674	974	2060
AtRoot569	LjRoot92	79.9893	863	2060
AtRoot569	LjRoot71	79.9884	614	2060
LjRoot281	LjRoot281	100	2045	2047
LjRoot281	AtRoot329	88.2611	1605	2047
LjRoot281	LjRoot277	88.1992	1596	2047
LjRoot281	AtRoot68	86.9334	1549	2047
LjRoot281	AtRoot71	86.9015	1538	2047
LjRoot281	LjRoot162	86.1066	1417	2047
LjRoot281	AtRoot562	86.0157	1476	2047
LjRoot281	LjRoot154	84.0576	1272	2047
LjRoot281	LjRoot59	84.0366	1274	2047
LjRoot281	AtRoot401	84.0248	1262	2047
LjRoot281	LjRoot54	83.9932	1270	2047
LjRoot281	AtRoot9	83.0574	1129	2047
LjRoot281	AtRoot569	82.9218	1118	2047
LjRoot281	LjRoot152	80.7901	962	2047
LjRoot281	LjRoot71	79.541	626	2047
LjRoot281	LjRoot92	79.4771	805	2047
LjRoot71	LjRoot71	100	1438	1441
LjRoot71	LjRoot92	81.3854	916	1441
LjRoot71	LjRoot152	80.3922	680	1441
LjRoot71	AtRoot9	80.1709	625	1441
LjRoot71	LjRoot154	80.0786	634	1441
LjRoot71	AtRoot401	80.0715	645	1441
LjRoot71	LjRoot59	80.0166	648	1441
LjRoot71	AtRoot71	80.0087	653	1441
LjRoot71	AtRoot68	80.0044	658	1441
LjRoot71	LjRoot54	80.0023	651	1441
LjRoot71	AtRoot569	79.9105	605	1441
LjRoot71	AtRoot329	79.8727	630	1441
LjRoot71	AtRoot562	79.8635	608	1441
LjRoot71	LjRoot162	79.7938	644	1441
LjRoot71	LjRoot277	79.7046	642	1441
LjRoot71	LjRoot281	79.6669	603	1441
AtRoot9	AtRoot9	100	2161	2165
AtRoot9	AtRoot569	89.6698	1664	2165
AtRoot9	AtRoot71	83.4727	1177	2165
AtRoot9	LjRoot154	83.4716	1163	2165
AtRoot9	AtRoot68	83.4646	1171	2165
AtRoot9	AtRoot401	83.4477	1185	2165
AtRoot9	LjRoot54	83.4317	1165	2165
AtRoot9	LjRoot59	83.4192	1169	2165
AtRoot9	LjRoot277	83.2356	1176	2165
AtRoot9	AtRoot329	83.1927	1220	2165
AtRoot9	AtRoot562	83.1488	1119	2165
AtRoot9	LjRoot281	83.0914	1133	2165
AtRoot9	LjRoot162	83.043	1126	2165
AtRoot9	LjRoot152	81.325	1016	2165
AtRoot9	LjRoot92	80.2955	886	2165
AtRoot9	LjRoot71	80.1125	653	2165

S4 Table cont.

query	reference	ANI	fragments_aligned	total_fragments
LjRoot92	LjRoot92	100	2254	2260
LjRoot92	LjRoot152	81.7635	1043	2260
LjRoot92	LjRoot71	81.3396	925	2260
LjRoot92	LjRoot59	80.4616	948	2260
LjRoot92	LjRoot54	80.4591	945	2260
LjRoot92	LjRoot154	80.4269	949	2260
LjRoot92	AtRoot9	80.4073	888	2260
LjRoot92	AtRoot401	80.3786	955	2260
LjRoot92	AtRoot68	80.2283	935	2260
LjRoot92	AtRoot71	80.2184	939	2260
LjRoot92	AtRoot569	80.028	861	2260
LjRoot92	LjRoot162	79.9115	869	2260
LjRoot92	AtRoot562	79.7335	790	2260
LjRoot92	AtRoot329	79.4861	877	2260
LjRoot92	LjRoot281	79.4773	803	2260
LjRoot92	LjRoot277	79.377	880	2260
LjRoot54	LjRoot54	100	2191	2192
LjRoot54	LjRoot59	99.9986	2185	2192
LjRoot54	LjRoot154	99.4953	2082	2192
LjRoot54	AtRoot401	96.5123	1933	2192
LjRoot54	AtRoot68	84.6346	1301	2192
LjRoot54	AtRoot71	84.633	1302	2192
LjRoot54	AtRoot329	84.4792	1386	2192
LjRoot54	LjRoot277	84.4644	1388	2192
LjRoot54	LjRoot162	84.1263	1232	2192
LjRoot54	LjRoot281	84.0899	1266	2192
LjRoot54	AtRoot562	83.9342	1257	2192
LjRoot54	AtRoot9	83.2155	1203	2192
LjRoot54	AtRoot569	83.182	1167	2192
LjRoot54	LjRoot152	81.6366	1010	2192
LjRoot54	LjRoot92	80.4417	956	2192
LjRoot54	LjRoot71	80.0587	643	2192
LjRoot162	LjRoot162	100	2242	2245
LjRoot162	AtRoot71	87.2025	1527	2245
LjRoot162	AtRoot68	87.1813	1515	2245
LjRoot162	AtRoot562	86.9773	1520	2245
LjRoot162	AtRoot329	86.6225	1514	2245
LjRoot162	LjRoot277	86.438	1516	2245
LjRoot162	LjRoot281	86.0993	1438	2245
LjRoot162	AtRoot401	84.2087	1205	2245
LjRoot162	LjRoot59	84.0947	1249	2245
LjRoot162	LjRoot154	84.0812	1250	2245
LjRoot162	LjRoot54	84.036	1256	2245
LjRoot162	AtRoot569	82.9536	1104	2245
LjRoot162	AtRoot9	82.9299	1137	2245
LjRoot162	LjRoot152	81.2992	1000	2245
LjRoot162	LjRoot92	80.0304	870	2245
LjRoot162	LjRoot71	79.8421	661	2245
AtRoot71	AtRoot71	100	2069	2070
AtRoot71	AtRoot68	99.9898	2060	2070
AtRoot71	LjRoot277	87.4352	1556	2070
AtRoot71	AtRoot562	87.3788	1526	2070
AtRoot71	AtRoot329	87.3179	1577	2070
AtRoot71	LjRoot162	87.2371	1513	2070
AtRoot71	LjRoot281	86.9956	1531	2070
AtRoot71	AtRoot401	84.6568	1307	2070
AtRoot71	LjRoot154	84.5391	1303	2070
AtRoot71	LjRoot59	84.5367	1324	2070
AtRoot71	LjRoot54	84.5278	1329	2070
AtRoot71	AtRoot9	83.3739	1186	2070
AtRoot71	AtRoot569	83.3303	1132	2070
AtRoot71	LjRoot152	81.657	1043	2070
AtRoot71	LjRoot92	80.1515	958	2070
AtRoot71	LjRoot71	79.9022	674	2070

S4 Table cont.

query	reference	ANI	fragments_aligned	total_fragments
LjRoot154	LjRoot154	100	2146	2146
LjRoot154	LjRoot54	99.5112	2075	2146
LjRoot154	LjRoot59	99.5079	2073	2146
LjRoot154	AtRoot401	96.4735	1917	2146
LjRoot154	AtRoot68	84.7001	1287	2146
LjRoot154	AtRoot71	84.6933	1284	2146
LjRoot154	LjRoot277	84.5598	1385	2146
LjRoot154	AtRoot329	84.4612	1403	2146
LjRoot154	LjRoot162	84.1939	1247	2146
LjRoot154	LjRoot281	84.1545	1253	2146
LjRoot154	AtRoot562	84.0197	1270	2146
LjRoot154	AtRoot9	83.2783	1181	2146
LjRoot154	AtRoot569	83.2084	1188	2146
LjRoot154	LjRoot152	81.7806	1002	2146
LjRoot154	LjRoot92	80.4374	968	2146
LjRoot154	LjRoot71	80.1289	643	2146
AtRoot562	AtRoot562	100	2058	2060
AtRoot562	AtRoot68	87.3892	1521	2060
AtRoot562	AtRoot71	87.3842	1519	2060
AtRoot562	LjRoot162	86.9913	1509	2060
AtRoot562	AtRoot329	86.6163	1488	2060
AtRoot562	LjRoot277	86.568	1483	2060
AtRoot562	LjRoot281	86.1546	1444	2060
AtRoot562	AtRoot401	84.216	1217	2060
AtRoot562	LjRoot154	84.1808	1216	2060
AtRoot562	LjRoot59	84.1031	1237	2060
AtRoot562	LjRoot54	84.0768	1238	2060
AtRoot562	AtRoot9	83.0327	1115	2060
AtRoot562	AtRoot569	82.7609	1102	2060
AtRoot562	LjRoot152	81.1599	963	2060
AtRoot562	LjRoot71	79.7896	617	2060
AtRoot562	LjRoot92	79.667	798	2060
LjRoot59	LjRoot59	100	2188	2190
LjRoot59	LjRoot54	99.9991	2186	2190
LjRoot59	LjRoot154	99.4982	2078	2190
LjRoot59	AtRoot401	96.4787	1938	2190
LjRoot59	AtRoot68	84.6832	1292	2190
LjRoot59	AtRoot71	84.6607	1298	2190
LjRoot59	AtRoot329	84.5092	1380	2190
LjRoot59	LjRoot277	84.4865	1389	2190
LjRoot59	LjRoot162	84.1435	1229	2190
LjRoot59	LjRoot281	84.1053	1265	2190
LjRoot59	AtRoot562	83.9366	1252	2190
LjRoot59	AtRoot9	83.2225	1199	2190
LjRoot59	AtRoot569	83.1575	1169	2190
LjRoot59	LjRoot152	81.6238	1004	2190
LjRoot59	LjRoot92	80.4463	954	2190
LjRoot59	LjRoot71	80.1126	636	2190
AtRoot401	AtRoot401	100	2307	2313
AtRoot401	LjRoot54	96.5006	1942	2313
AtRoot401	LjRoot59	96.4942	1940	2313
AtRoot401	LjRoot154	96.4683	1922	2313
AtRoot401	AtRoot71	84.5816	1305	2313
AtRoot401	AtRoot68	84.5781	1313	2313
AtRoot401	AtRoot329	84.398	1378	2313
AtRoot401	LjRoot277	84.3979	1370	2313
AtRoot401	LjRoot281	84.1623	1231	2313
AtRoot401	LjRoot162	84.1557	1220	2313
AtRoot401	AtRoot562	84.1227	1233	2313
AtRoot401	AtRoot9	83.2528	1177	2313
AtRoot401	AtRoot569	83.1742	1183	2313
AtRoot401	LjRoot152	81.6725	1011	2313
AtRoot401	LjRoot92	80.4894	946	2313
AtRoot401	LjRoot71	80.0401	651	2313

S4 Table cont.

query	reference	ANI	fragments_aligned	total_fragments
AtRoot329	AtRoot329	100	2213	2216
AtRoot329	LjRoot277	95.6385	1859	2216
AtRoot329	LjRoot281	88.1927	1621	2216
AtRoot329	AtRoot71	87.3016	1556	2216
AtRoot329	AtRoot68	87.2535	1570	2216
AtRoot329	AtRoot562	86.6392	1504	2216
AtRoot329	LjRoot162	86.6319	1509	2216
AtRoot329	LjRoot154	84.5684	1383	2216
AtRoot329	LjRoot59	84.5158	1389	2216
AtRoot329	LjRoot54	84.4924	1397	2216
AtRoot329	AtRoot401	84.4618	1376	2216
AtRoot329	AtRoot9	83.06	1216	2216
AtRoot329	AtRoot569	83.0503	1205	2216
AtRoot329	LjRoot152	80.8768	1018	2216
AtRoot329	LjRoot71	79.8262	640	2216
AtRoot329	LjRoot92	79.5913	867	2216
LjRoot277	LjRoot277	100	2173	2177
LjRoot277	AtRoot329	95.5843	1853	2177
LjRoot277	LjRoot281	88.1675	1576	2177
LjRoot277	AtRoot68	87.3666	1553	2177
LjRoot277	AtRoot71	87.3517	1550	2177
LjRoot277	AtRoot562	86.5025	1481	2177
LjRoot277	LjRoot162	86.4155	1507	2177
LjRoot277	LjRoot154	84.4705	1380	2177
LjRoot277	LjRoot59	84.4339	1398	2177
LjRoot277	LjRoot54	84.4292	1398	2177
LjRoot277	AtRoot401	84.3419	1366	2177
LjRoot277	AtRoot9	83.068	1193	2177
LjRoot277	AtRoot569	83.0213	1178	2177
LjRoot277	LjRoot152	80.7215	993	2177
LjRoot277	LjRoot71	79.7369	637	2177
LjRoot277	LjRoot92	79.3883	871	2177
LjRoot152	LjRoot152	100	1923	1924
LjRoot152	AtRoot401	81.8327	994	1924
LjRoot152	LjRoot154	81.7671	989	1924
LjRoot152	LjRoot92	81.7404	1054	1924
LjRoot152	LjRoot54	81.7097	1004	1924
LjRoot152	LjRoot59	81.7003	1004	1924
LjRoot152	AtRoot71	81.6684	1034	1924
LjRoot152	AtRoot68	81.6465	1026	1924
LjRoot152	AtRoot9	81.3693	1019	1924
LjRoot152	LjRoot162	81.3213	1003	1924
LjRoot152	AtRoot569	81.2781	990	1924
LjRoot152	AtRoot562	81.1311	964	1924
LjRoot152	AtRoot329	80.938	1009	1924
LjRoot152	LjRoot281	80.8208	960	1924
LjRoot152	LjRoot277	80.7627	984	1924
LjRoot152	LjRoot71	80.4955	678	1924
AtRoot68	AtRoot68	100	2071	2072
AtRoot68	AtRoot71	99.9885	2060	2072
AtRoot68	AtRoot562	87.3899	1526	2072
AtRoot68	LjRoot277	87.3674	1559	2072
AtRoot68	AtRoot329	87.2578	1588	2072
AtRoot68	LjRoot162	87.2336	1511	2072
AtRoot68	LjRoot281	86.9827	1546	2072
AtRoot68	LjRoot154	84.6868	1286	2072
AtRoot68	LjRoot59	84.6794	1301	2072
AtRoot68	LjRoot54	84.6394	1311	2072
AtRoot68	AtRoot401	84.6194	1287	2072
AtRoot68	AtRoot9	83.3816	1186	2072
AtRoot68	AtRoot569	83.3629	1136	2072
AtRoot68	LjRoot152	81.6084	1037	2072
AtRoot68	LjRoot92	80.1976	950	2072
AtRoot68	LjRoot71	79.9539	672	2072

Kruskal-Wallis rank sum test followed by Wilcoxon rank sum test with Bonferroni p-value adjustment revealed no significant difference between ANI to R401 of protective vs. non-protective strains

> when strains used as query for the FastANI analysis: $p = 1$

> when strains used as reference for the FastANI analysis: $p = 0.85$
