# Supplementary material for: Pseudomonas intra-genus competition determines the protective function of synthetic bacterial communities in Arabidopsis thaliana
Source: PLoS Biol. 2025 Jul 15;23(7):e3002882. doi: 10.1371/journal.pbio.3002882 (PMC12262851; doi:10.1371/journal.pbio.3002882)
Supplement: S5 Table — (PDF) [file pbio.3002882.s020.pdf]

S5 Table: Growth parameters of individual *Pseudomonas* isolates.

Maximum growth rate and doubling time of strains grown in minimal medium supplemented with artifical root exudates (ARE; nine carbon sources, see Materials and Metho

Protection *in planta* is based on the plant phenotype after co-cultivation with individual strains and infection with R401wt.

strain	max. growth rate per hour	R^2	doubling time (h)	protection in planta
LjRoot152	0.625083948	0.99567509	1.10865109	TRUE
LjRoot154	0.993691875	0.99800832	0.697399282	TRUE
LjRoot162	0.632616148	0.99580937	1.095451013	FALSE
LjRoot277	0.827388013	0.99648532	0.837575586	TRUE
LjRoot281	0.838181624	0.98725226	0.826789779	TRUE
LjRoot54	0.53308627	0.99897171	1.299977206	TRUE
LjRoot59	0.712068474	0.9955399	0.973221011	TRUE
LjRoot71	0.689325654	0.99615484	1.005330349	TRUE
LjRoot92	1.146934156	0.99570306	0.604219516	FALSE
R401mut	0.788178893	0.99839107	0.879242017	n.a.
R401wt	0.576376674	0.99948148	1.202338733	n.a.
AtRoot329	0.490102234	0.99943194	1.413990698	TRUE
AtRoot562	0.944077582	0.99668525	0.734049842	FALSE
AtRoot569	0.685330897	0.99938348	1.011190365	TRUE
AtRoot68	0.604928751	0.99312951	1.145589457	FALSE
AtRoot71	0.68928678	0.9978436	1.005387046	FALSE
AtRoot9	0.700540611	0.9862462	0.989236011	TRUE

Kruskal-Wallis rank sum test followed by Wilcoxon rank sum test with Bonferroni p-value adjustment revealed no significant difference between growth rates of protective vs. non-protective strains ($p = 0.76$).
