# Supplementary material for: Pseudomonas intra-genus competition determines the protective function of synthetic bacterial communities in Arabidopsis thaliana
Source: PLoS Biol. 2025 Jul 15;23(7):e3002882. doi: 10.1371/journal.pbio.3002882 (PMC12262851; doi:10.1371/journal.pbio.3002882)
Supplement: S6 Table — (PDF) [file pbio.3002882.s021.pdf]

S6 Table: Number of catabolic gene clusters for the catabolisms of the indicated carbon sources per bacterial strain detected by rhizoSMASH (https://git.wur.nl/rhizosmash).

Strain	3-hydroxyphenylpropanoate/2-oxopentenoate	3-oxoadipate	3-oxoadipate/protocatchuate/quinate/shikimate	4-hydroxylphenylacetate	anthranilate	benzoate	benzoate/catachol/2-oxopentenoate	catechol	D-lysine	D-pipecolate	d-threonate	delta-aminovalerate	gamma-aminobutyrate	glutamine	glutarate	indole-3-acetate	inositol
Root401	0	1	2	1	0	1	1	1	0	1	0	1	0	1	1	0	1
LjRoot152	0	0	2	1	0	1	1	1	1	1	0	1	0	1	1	0	0
LjRoot154	0	1	2	1	0	1	1	1	0	1	0	1	0	1	1	0	1
LjRoot162	2	1	3	1	0	1	3	1	0	1	0	1	0	1	1	1	0
LjRoot277	0	1	1	0	0	1	1	1	0	1	0	1	0	1	2	0	1
LjRoot281	0	0	2	0	0	1	1	1	0	1	1	1	0	1	1	1	0
LjRoot54	0	1	2	1	0	1	1	1	0	1	0	1	0	1	1	0	1
LjRoot59	0	1	2	1	0	1	1	1	0	1	0	1	0	1	1	0	1
LjRoot71	0	0	0	0	0	0	0	0	0	0	0	1	1	1	1	0	0
LjRoot92	2	1	2	1	0	1	3	1	0	1	0	1	1	1	1	1	0
AtRoot329	1	0	1	1	0	1	2	1	0	1	1	1	0	1	1	0	1
AtRoot562	0	1	2	0	0	1	1	1	0	1	0	1	0	1	1	2	0
AtRoot569	0	0	1	1	1	0	0	1	0	1	0	1	0	1	1	0	1
AtRoot68	1	0	1	1	0	1	2	1	0	1	0	1	0	1	2	1	0
AtRoot71	1	0	1	1	0	1	2	1	0	1	0	1	0	1	2	1	0
AtRoot9	0	0	1	1	1	0	0	1	0	1	0	1	0	1	1	0	1

S6 Table cont.

Strain	L-lysine	L-pipecolate	L-proline	l-threonate	nicotinate	phenylacetate	phenylethylamine	polyamine	protocatchuate	quinate/shikimate	salicylate/catechol/2-oxopentenoate	toluene	trehalose	tyramine/dopamine/4HPA	tyramine/phenylethylamine	vanillate	xylose
Root401	1	1	1	0	0	0	0	1	1	1	0	0	2	1	0	0	1
LjRoot152	1	1	1	0	0	1	1	3	0	1	0	0	0	1	1	0	0
LjRoot154	1	1	1	0	0	0	1	1	1	1	0	0	2	1	0	0	1
LjRoot162	1	1	1	1	0	1	1	4	3	1	2	1	0	1	1	1	0
LjRoot277	1	1	1	1	1	1	0	4	0	1	0	0	1	0	0	0	0
LjRoot281	1	1	1	0	0	1	1	4	0	0	0	0	0	0	1	0	0
LjRoot54	1	1	1	0	0	0	1	1	1	1	0	0	2	1	0	0	1
LjRoot59	1	1	1	0	0	0	1	1	1	1	0	0	2	1	0	0	1
LjRoot71	0	0	0	0	0	0	0	1	0	0	0	0	0	0	0	0	0
LjRoot92	1	1	0	0	0	1	1	2	3	1	2	0	0	1	1	0	0
AtRoot329	1	1	1	1	1	1	0	4	2	1	1	0	1	2	0	0	0
AtRoot562	1	1	1	0	0	1	1	4	0	1	0	0	0	0	1	0	0
AtRoot569	1	1	1	0	1	0	0	2	0	0	0	0	1	1	0	0	1
AtRoot68	1	1	1	0	0	1	1	3	1	1	1	0	0	1	1	0	0
AtRoot71	1	1	1	0	0	1	1	3	1	1	1	0	0	1	1	0	0
AtRoot9	1	1	1	0	0	0	0	2	1	0	0	0	1	1	0	0	1
