# Supplementary material for: Pseudomonas intra-genus competition determines the protective function of synthetic bacterial communities in Arabidopsis thaliana
Source: PLoS Biol. 2025 Jul 15;23(7):e3002882. doi: 10.1371/journal.pbio.3002882 (PMC12262851; doi:10.1371/journal.pbio.3002882)
Supplement: S7 Table — (PDF) [file pbio.3002882.s022.pdf]

S7 Table: Euclidean distances of R401 to the other *Pseudomonas* strains based on the presence of catabolic gene clusters listed in S6 Table.
Protection is based on the plant phenotype after co-cultivation with individual strains and infection with R401wt.

Strain	Distance_to_Root401	protection
Root401	0	na
LjRoot154	1	TRUE
LjRoot54	1	TRUE
LjRoot59	1	TRUE
AtRoot9	2.828427125	TRUE
AtRoot569	3.16227766	TRUE
LjRoot152	4	TRUE
LjRoot277	4.358898944	TRUE
AtRoot68	4.472135955	FALSE
AtRoot71	4.472135955	FALSE
AtRoot329	4.69041576	TRUE
LjRoot71	4.795831523	TRUE
LjRoot281	5	TRUE
AtRoot562	5	FALSE
LjRoot92	5.385164807	FALSE
LjRoot162	6.244997998	FALSE

Kruskal-Wallis rank sum test followed by Wilcoxon rank sum test with Bonferroni p-value adjustment revealed a significant difference between distance values of protective vs. non-protective strains (p = 0.027).

Pearson's product-moment correlation

data: distances and protective_property2
t = -2.4541, df = 13, p-value = 0.02899
alternative hypothesis: true correlation is not equal to 0
95 percent confidence interval:
 -0.83442412 -0.07082364
sample estimates:
      cor
 -0.5626725
